# Subspecialization of R2R3-MYB Repressors for Anthocyanin and Proanthocyanidin Regulation in Forage Legumes

**DOI:** 10.3389/fpls.2015.01165

**Published:** 2015-12-23

**Authors:** Nick W. Albert

**Affiliations:** AgResearch LimitedPalmerston North, New Zealand

**Keywords:** anthocyanin, condensed tannin, flavonoid, proanthocyanidin, repressor, transcription factor

## Abstract

The synthesis of anthocyanin pigments and proanthocyanidins (condensed tannins) is regulated by MYB-bHLH-WDR (MBW) transcription factor complexes in all angiosperms studied to date. *Tr-MYB133* and *Tr-MYB134* were isolated from *Trifolium repens* and encode R2R3-MYBs that antagonize the activity of MBW activation complexes. These two genes are conserved in other legume species, and form two sub-clades within the larger anthocyanin/proanthocyanidin clade of MYB repressors. However, unlike petunia and *Arabidopsis*, these R2R3-MYB repressors do not prevent ectopic accumulation of anthocyanins or proanthocyanidins. Instead, they are expressed when anthocyanins or proanthocyanidins are being synthesized, and provide feedback regulation to MBW complexes. This feedback occurs because *Tr-MYB133* and *Tr-MYB134* are themselves regulated by MBW complexes. *Tr-MYB133* is regulated by MBW complexes containing anthocyanin-related R2R3-MYB proteins (Tr-RED LEAF), while *Tr-MYB134* is regulated by complexes containing the proanthocyanidin R2R3-MYBs (Tr-MYB14). Other features of the MBW gene regulation networks are also conserved within legumes, including the ability for the anthocyanin MBW complexes to activate the expression of the AN1/TT8 clade bHLH factor. The regulation of Tr-MYB133 and Tr-MYB134 by distinct, pathway-specific MBW complexes has resulted in subspecialization for controlling anthocyanin or proanthocyanidin synthesis.

## Introduction

Anthocyanins and proanthocyanidins (syn. condensed tannins) are related flavonoid compounds that are produced by plants throughout development, and in response to biotic and abiotic stresses (Dixon and Paiva, 1995). Anthocyanins are red/purple/blue pigments that provide color to flowers, fruits and vegetative tissues in plants, fulfilling a variety of physiological requirements. In flowers and fruits, anthocyanins provide visual cues to pollinators and seed distributers, respectively, and can form highly elaborate patterns (e.g., stripes, spots), especially when combined with other types of pigments (Davies et al., 2012). In vegetative tissues, anthocyanins are commonly produced in response to environmental conditions that may compromise photosynthetic activity (e.g., high light, cold, nutrient deficiency), which can result in photoinhibition and the production of reactive oxygen species (ROS; Gould, 2004). Anthocyanins ameliorate the effect of these stresses by screening underlying photosynthetic tissue from excess light (Neill and Gould, 2003; Hughes et al., 2005; Albert et al., 2009; Hatier et al., 2013), and may also act as antioxidants *in planta* to neutralize ROS (Gould et al., 2002; Landi et al., 2014). Proanthocyanidins are structurally related to anthocyanins, being composed of anthocyanidin-based subunits (flavan-3-ols) that are polymerized into proanthocyanidin molecules (Zhao et al., 2010). The accumulation and distribution of proanthocyanidins varies widely between plant species, being found in seed coats of most angiosperms, but also accumulating in flowers, fruits, and leaves in some taxa (Dixon et al., 2005). They act as feeding deterrents for chewing insects and herbivores because of their astringency properties, and may also have structural functions (Barbehenn and Constabel, 2011).

Anthocyanins and proanthocyanidins offer health benefits to humans and other animals. Dietary anthocyanins have been associated with improvements in a range of risk factors for chronic diseases including metabolic syndrome, cardiovascular disease, and certain cancers (Martin et al., 2011), and there is increasing interest in fortifying fruits and vegetables with increased quantities of anthocyanins (Butelli et al., 2008; Espley et al., 2013). In contrast, proanthocyanidins offer a range of health and production benefits to grazing ruminants (e.g., sheep, cattle), due to their protein-binding properties. Dietary protein from forage legumes is rapidly fermented in the rumen, producing methane gas within stable foams and resulting in the potentially lethal condition, pasture bloat. The inclusion of proanthocyanidins in the diet prevents pasture bloat, by protecting protein from premature fermentation. This increases animal weight and milk production because of improved amino acid absorption, and also reduces methane and nitrogen emissions (Douglas et al., 1999; Beauchemin et al., 2007). The major forage legumes grown for pastoral agriculture are alfalfa (*Medicago sativa*) and white clover (*Trifolium repens*), which lack significant quantities of proanthocyanidins in their leaves. Therefore, there is strong interest in developing high-proanthocyanidin forage legumes using genetic technologies (Dixon et al., 2013).

Anthocyanin and proanthocyanidin biosynthesis is regulated primarily at the transcriptional level, by MBW transcription factor complexes (Koes et al., 2005). Both anthocyanin and proanthocyanidin biosynthesis share the same WDR protein and bHLH2/AN1/TT8 clade bHLH factor, which form the MBW complex together with R2R3-MYB proteins (Zhang et al., 2003; Baudry et al., 2004; Davies et al., 2012). These R2R3-MYB proteins provide specificity to the complex and determine which pathways are regulated (anthocyanins vs. proanthocyanidins; Ramsay and Glover, 2005; Heppel et al., 2013). While all three components are necessary to activate anthocyanin synthesis, the R2R3-MYB genes largely determine the pigmentation patterning, as these are typically encoded by small gene families with diverse spatial expression patterns (Schwinn et al., 2006; Albert et al., 2011; Lowry et al., 2012). For proanthocyanidin regulation, multiple R2R3-MYB factors have been identified that act within MBW complexes. However, unlike the small gene families of anthocyanin-related R2R3-MYB genes, the proanthocyanidin-related MYBs studied to date appear to differ in their functions. For example, *MYB14* from *Trifolium arvense* and *Medicago truncatula* activates the accumulation of proanthocyanidins when expressed in *T. repens* or *Medicago* (Hancock et al., 2012; Liu et al., 2014), while other proanthocyanidin-related MYBs are either less effective or are unable to activate proanthocyanidin synthesis when expressed alone (Sharma and Dixon, 2005; Verdier et al., 2012). It is not yet clear how these MYB genes act together, although it seems likely that they may act upon different subsets of target genes, to cooperatively activate proanthocyanidin synthesis (Deluc et al., 2008; Terrier et al., 2009; Liu et al., 2014).

The MBW complex operates within a gene regulation network that involves reinforcement and feedback repression. Current models suggest that the MBW complex activates the expression of the *bHLH2/AN1/TT8* clade bHLH factor to provide reinforcement, and also activates the expression of *R2R3-MYB* and *R3-MYB* repressors to provide feedback repression (Albert et al., 2014a). In both petunia and *Arabidopsis*, the R2R3-MYB repressors (At-MYBL2 contains a partial R2 domain) are also expressed in leaves during non-stress conditions, preventing inappropriate synthesis of anthocyanins (Dubos et al., 2008; Albert et al., 2011). The R2R3-MYB repressors actively repress transcription through motifs (EAR, TLLLFR) in their C-terminal domains (Aharoni et al., 2001; Matsui et al., 2008; Albert et al., 2014a), while the small R3-MYB proteins act competitively to inhibit the formation of functional MBW complexes (Schellmann et al., 2002; Yuan et al., 2013; Albert et al., 2014a).

The R2R3-MYB repressors that regulate anthocyanin (Aharoni et al., 2001; Dubos et al., 2008; Matsui et al., 2008; Albert et al., 2011, 2014a; Salvatierra et al., 2013) and proanthocyanidin synthesis (Huang et al., 2014; Cavallini et al., 2015; Yoshida et al., 2015) act upon MBW complexes (Matsui et al., 2008; Albert et al., 2014a), which differs from the R2R3-MYB repressors that were first identified as regulators of cinnamic acid derivatives (e.g., At-MYB4; Tamagnone et al., 1998; Jin et al., 2000). Many features of the MBW gene regulation network are likely to be conserved in eudicots (Albert et al., 2014a), if not more widely across angiosperms (Albert et al., 2014b), but it is not yet known which features are conserved and which differ in legumes.

The synthesis of anthocyanins and proanthocyanidins in white clover (*T. repens*) is tightly regulated, forming intricate anthocyanin pigmentation patterns in leaves (Albert et al., 2015) and restricted accumulation of proanthocyanidins to specific organs and tissues (Abeynayake et al., 2012; Hancock et al., 2012). Improving the content and distribution of proanthocyanidins in white clover is highly desirable for agricultural purposes, but this first requires an understanding of how the gene regulation networks determine anthocyanin and proanthocyanidin synthesis. The roles of repressors for proanthocyanidin regulation in forage legumes are currently unknown, yet these have been proposed as interesting gene targets for increasing the anthocyanin or proanthocyanidin content through mutagenesis and/or breeding strategies (Albert et al., 2014c).

The aim of this research was to investigate how the anthocyanin and proanthocyanidin biosynthetic pathways are regulated in legumes, and determine if R2R3-MYB repressors perform similar roles for regulating flavonoid synthesis, as they do in other groups of angiosperms. In this study, two distinct R2R3-MYB repressors were identified and characterized in white clover for their roles for regulating anthocyanin and proanthocyanidin synthesis.

## Materials and Methods

### Plant Material and Growth Conditions

*Trifolium repens* cultivar ‘Sustain’ plants were germinated from seed while *T. repens* plants with the ‘red leaf’ or ‘red leaflet’ anthocyanin leaf markings were clonally propagated (four biological replicates). Plants were grown in pots within a greenhouse that was heated at 15°C and vented at 25°C with ambient lighting. Clonal copies of plants with the ‘red leaflet’ marking were also grown outside during winter [July 2013; mean monthly air temperature 6.1°C/9.7°C (min/max); mean min ground temperature 3.3°C; mean relative humidity 88.7%], to expose plants to cold temperatures that induce the anthocyanin leaf marking (Albert et al., 2015). Expanding leaves were sampled for RNA isolation early during the illuminated part of the day, while temperatures will still cool outside (>10°C). Inflorescences were sampled from the ‘red leaflet’ genotype grown in the greenhouse at two developmental stages; when the first flowers were beginning to open (Inf. A), and when half of the inflorescence had open flowers, but before any flowers senesced (Inf. B). Tissue was immediately frozen in liquid nitrogen upon collection. The presence of proanthocyanidins were detected by staining with *p*-dimethylaminocinnamaldehyde (DMACA; Li et al., 1996).

### RNA Isolation

Total RNA was isolated from ∼100 mg leaf tissue with the ISOLATE II RNA Plant mini kit using lysis buffer APR (Bioline, Auckland, New Zealand). The presence of proanthocyanidins in inflorescences required modification of the cell lysis conditions, to prevent proanthocyanidins from binding nucleic acids and inhibiting RNA isolation and purification. Frozen tissue (∼100 mg) was immediately homogenized in lysis buffer BPR supplemented with 4% (w/v) cetyltrimethyl ammonium bromide and 4% (w/v) polyvinylpyrrolidone, and then processed as per manufacturer’s instructions.

### Gene Isolation

First stand cDNA was synthesized from 4 μg total RNA (mixed tissues and developmental stages from leaves and inflorescences) using Tetro cDNA synthesis kit (Bioline), primed with NAg11 (**Supplementary Table S1**). PCR using degenerate oligonucleotides (NAg3/NAg4) was performed using 2 μL of first strand cDNA, 250 nM of each oligonucleotide with MyTaq^TM^ Red polymerase mastermix (Bioline) in 50 μL reactions. Amplification was performed with the following cycling conditions: 94°C 2 min; (94°C 15 s, 58-1°C/cycle 30 s, 72°C 1 min) × 10; (94°C 15 s, 48°C 30 s, 72°C 1 min) × 30; 72°C 5 min, 12°C hold. PCR products were sequenced, and primers were designed to extend sequences by 3′ RACE. 3′ RACE PCR was performed upon cDNAs primed with NAg11, essentially as described in Albert et al. (2015); primers NAg34/NAg12, and NAg35/NAg13 were used for the primary and secondary PCR reactions, respectively, for Tr-MYB133; primers NAg72/NAg12, and NAg73/NAg13 were used for the primary and secondary PCR reactions, respectively, for Tr-MYB134. The sequences were extended further by 5′ RACE using the SMARTer^®^ RACE cDNA amplification kit (Clontech), following the manufacturer’s instructions, with gene-specific primers: Tr-MYB133, NAg49 and NAg47; Tr-MYB134, NAg45, and NAg46. Full cDNAs were amplified with primers NAg100/NAg103 and NAg72/NAg96 for *Tr-MYB133* and *Tr-MYB134*, respectively.

### Phylogenetic Analysis

A maximum likelihood phylogenetic tree was generated, with 1000 bootstrap replicates, upon amino acid sequences for R2R3-MYB repressors which were aligned using MUSCLE in Geneious software (v7; Kearse et al., 2012) and manually adjusted.

### Quantitative Reverse Transcription-Polymerase Chain Reaction (qRT-PCR)

First strand cDNA was prepared from 1 μg DNAseI-treated total DNA using iScript reverse transcriptase mastermix (Biorad), and diluted 20-fold with water. Quantitative reverse transcription-polymerase chain reaction (qRT-PCR) was performed essentially as described in Dorling et al. (2011) upon four biological replicates per treatment, with three technical replicates, using Kapa SYBR^®^ FAST Universal qPCR reagents for *Tr-MYB133*, *Tr-MYB134*, *Tr-AN1*, *DFR*, *GAPDH*, and *PP2* assays (KAPA Biosystems) or Kapa probe FAST Universal qPCR reagents for *ACTIN, ANR*, and *Tr-MYB14* (**Supplementary Table S1** for primer and probe sequences). Relative transcript abundance was determined relative to the geometric mean of *ACTIN*, *GAPDH*, and *PP2* (Dorling et al., 2011).

### Luciferase Assays

The *ANR*, *Tr-MYB133*, and *Tr-MYB134* promoters from *T. repens* were amplified and cloned into a dual luciferase vector, pNWA62, as has previously been done for the *DFR* promoter (Albert et al., 2015). Effector constructs that constitutively express transcription factors from a *CaMV35S* promoter were generated by cloning the coding sequence into pENTR-D Topo, and LR-recombination (Life technologies) into the gateway adapted binary vector pRSH1. Binary vectors containing the effector and dual luciferase reporter constructs were transformed into *Agrobacterium tumefaciens* (GV3101). *Agrobacterium* strains were grown and cells were resuspended in 10 mM MgCl_2_ containing 250 μM acetosyringone and cultured for 4 h at room temperature, and cultures were infiltrated into *Nicotiana benthamiana* leaves. Dual luciferase assays were performed with six biological replicates, using DLAR-2B reagents (Targeting Systems) as described in Albert et al. (2014a). Data are expressed as ratios of firefly:renilla luciferase activity.

### Statistical Analyses

One-way ANOVA was performed with *post hoc* Fishers LSD (5%) using Genstat software (version 15). Analyses were performed upon log_10_-transformed data to normalize variances.

## Results

### Two Distinct Clades of Flavonoid R2R3-MYB Repressors Exist in Legumes

**FIGURE 1:**
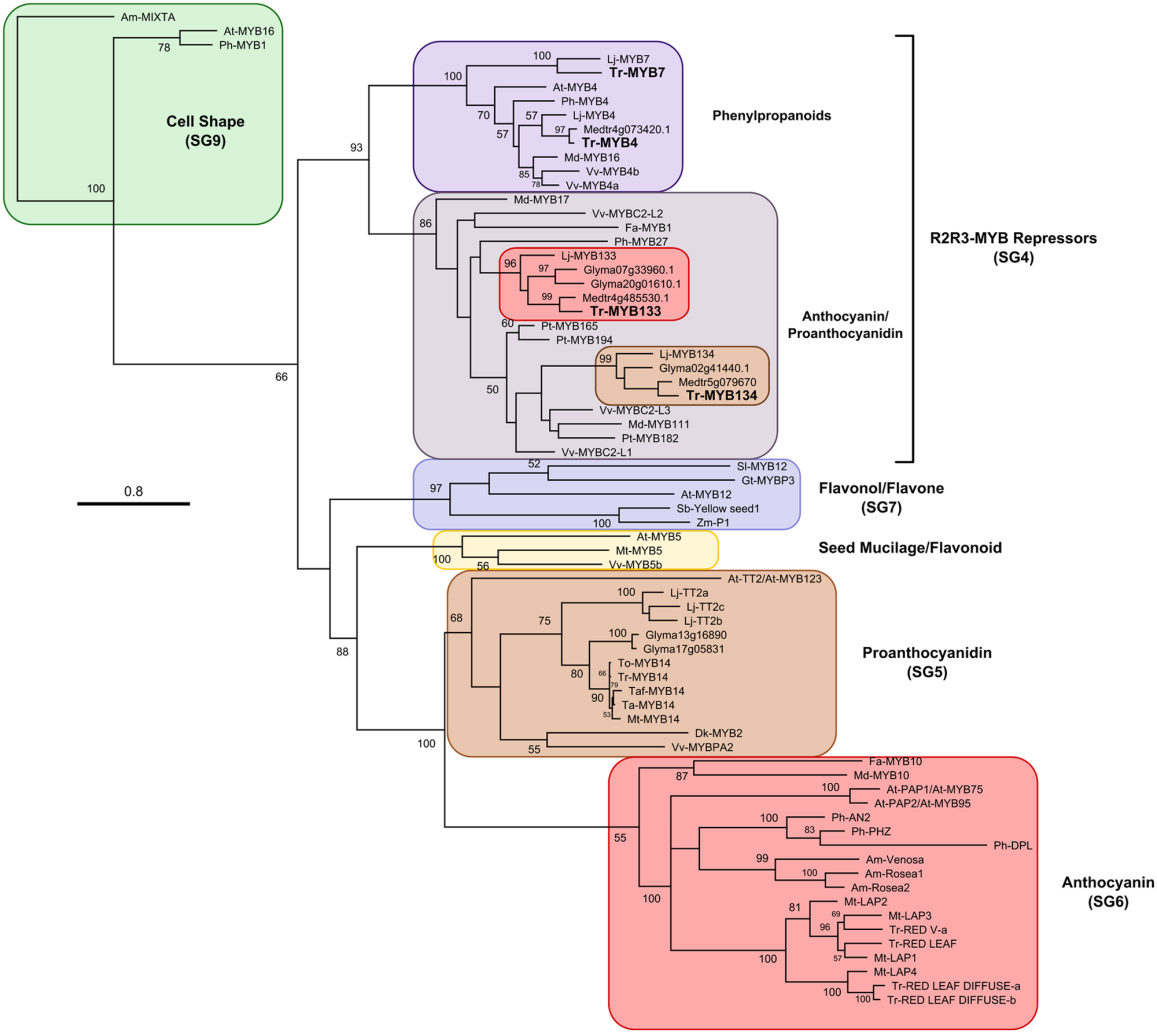
**Maximum likelihood phylogenetic tree of R2R3-MYB proteins.** Nodes with bootstrap support >50% from 1000 replicates are shown. Accession numbers for sequences used to construct this tree are provided in **Supplementary Table S2**. *At, Arabidopsis thaliana; Am, Antirrhinum majus; Fa, Fragaria ananassa; Gt, Gentiana triflora; Gm, Glycine max; Lj, Lotus japonicus; Md, Malus domestica; Mt, Medicago truncatula; Ph, Petunia hybrida; Pt, Populus trichocarpa; Sl, Solanum lycopersicum; Ta, Trifolium arvense; Taf, T. affine; Tr, T. repens; To, T. occidentale; Vv, Vitis vinifera.*

A candidate gene approach was taken to identify R2R3-MYB repressor genes that may be associated with regulating anthocyanin and/or proanthocyanidin synthesis in white clover. *Fa-MYB1* from strawberry (Aharoni et al., 2001), *Ph-MYB27* from petunia (Albert et al., 2011, 2014a) and *At-MYBL2* (a truncated R2R3-MYB repressor) from *Arabidopsis* (Dubos et al., 2008; Matsui et al., 2008) were used as queries to search sequence databases for *Lotus japonicus*, *M. Truncatula*, and *Glycine max* using BLASTp. Criteria used for screening putative repressors were the presence of the bHLH interaction motif ([D/E]Lx_2_[R/K]x_3_Lx_6_Lx_3_R; Zimmermann et al., 2004) and either an EAR motif (LxLxL, DLNxxP; Kagale et al., 2010) or similarity to the TLLLFR repression motif present in At-MYBL2 (Matsui et al., 2008). The putative repressors identified from *Lotus* (Lj-MYB133, Lj-MYB134), *Medicago* (Medtr4g85530, Medtr5g079670), and *Glycine* (Glyma20g01610, Glyma07g33960, Glyma02g41440) fall into two well-supported clades, represented by Lj-MYB133 and Lj-MYB134 from *Lotus* (**Figure 1**; **Supplementary Figure S1**). These sequences were aligned, and degenerate oligonucleotides were designed to the conserved DNA binding domain.

Two genes putatively encoding R2R3-MYB repressors were isolated from *T. repens* using a combination of PCR with degenerate oligonucleotides, and 3′ and 5′ rapid amplification of cDNA ends (RACE). The genes were named *Tr-MYB133* and *Tr-MYB134*, based upon their sequence similarity to the corresponding homologs from *L. japonicus* (Shelton et al., 2012). Both Tr-MYB133 and Tr-MYB134 have the bHLH interaction motif present in the MYB DNA binding domain, and contain an LxLxL-type EAR repression motif in the C-terminus (Tr-MYB133 LDLNLELSL; Tr-MYB134 LNLEL).

Subsequent to isolating *Tr-MYB133* and *Tr-MYB134*, an RNA-seq dataset became available from *T. repens* leaf tissue. Two additional R2R3-MYB repressors were identified, *Tr-MYB4* and *Tr-MYB7*. These sequences belong to the MYB4-like clade of repressors, which are involved in regulating phenylpropanoid metabolism (e.g., cinnamic acid derivatives), rather than anthocyanins/proanthocyanidins directly, and therefore these were not pursued further.

The sequences of the putative R2R3-MYB repressors from *T. repens* were compared with other R2R3-MYB repressors and used to construct a phylogenetic tree (**Figure 1**). Tr-MYB133 and Tr-MYB134 fall within the well-supported clade of anthocyanin and proanthocyanidin repressors, which includes the functionally characterized anthocyanin repressors Fa-MYB1 and Ph-MYB27, and the proanthocyanidin repressors Vv-MYBC2-L1 and Pt-MYB165. The legume sequences form two distinct subclades, separating Tr-MYB133 and Tr-MYB134, raising the possibility that these two genes may have subspecialized, at least within legumes.

### Tr-MYB133 and Tr-MYB134 Repress MBW Complex Activity

The activities of *Tr-MYB133* and *Tr-MYB134* were investigated by promoter activation/repression assays, using *Agrobacterium*-infiltrated *N. benthamiana* leaves. The promoters for *DIHYDROFLAVONOL 4-REDUCTASE* (*DFR*), a biosynthetic gene common to anthocyanin and proanthocyanidin pathways, and the proanthocyanidin biosynthetic gene *ANTHOCYANIDIN REDUCTASE* (*ANR*) were isolated from *T. repens*, and cloned into a dual luciferase reporter construct. Effector constructs expressing *Tr-MYB133* or *Tr-MYB134* were assayed with combinations of MBW constructs that have been characterized previously; the proanthocyanidin regulator *Ta-MYB14* from *Trifolium arvense* (94% amino acid identity to Tr-MYB14; Hancock et al., 2012), the anthocyanin activator *Tr-RED LEAF* and bHLH factor *Tr-AN1* (Albert et al., 2015).

**FIGURE 2:**
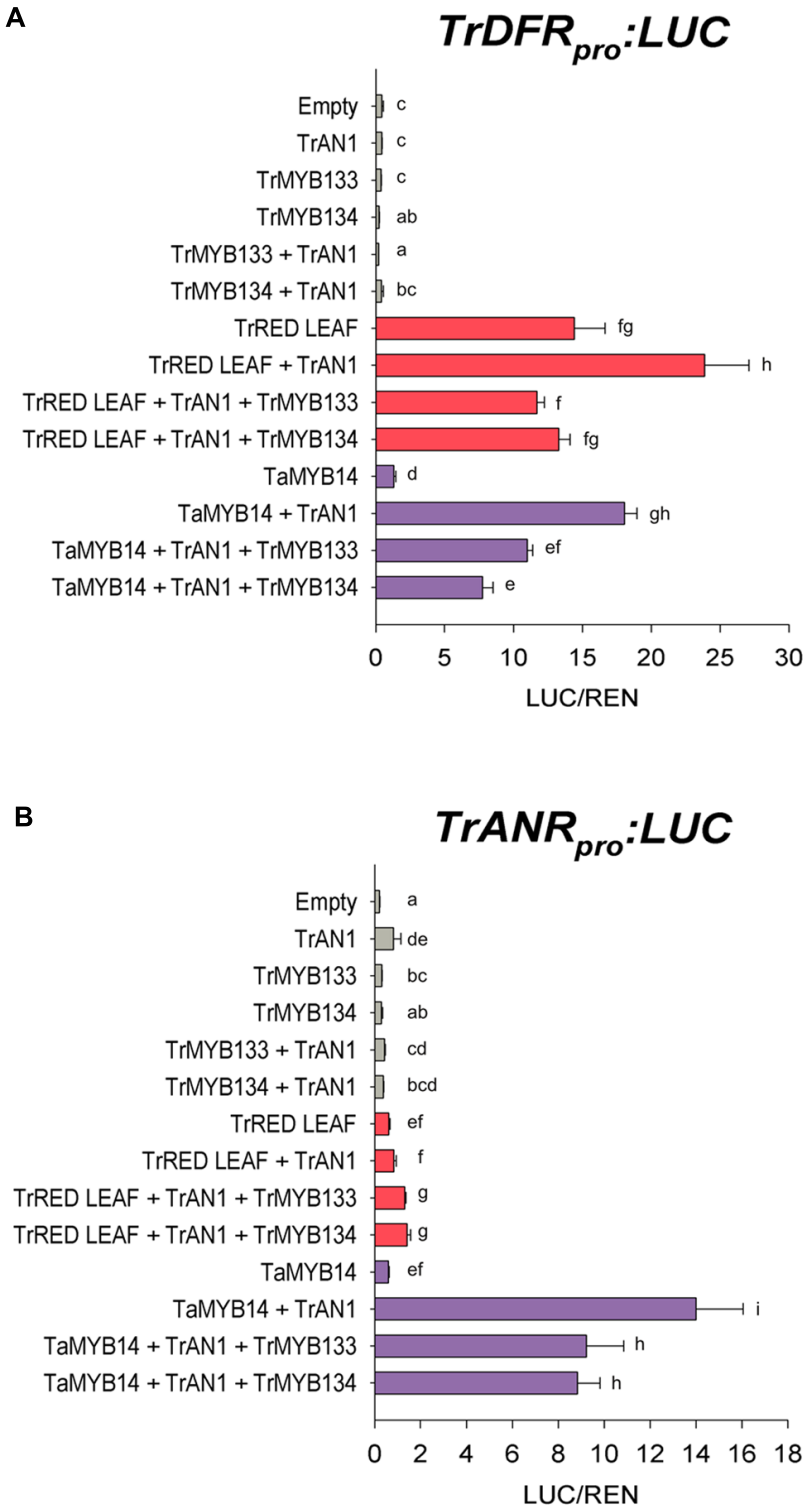
***Tr-MYB133* and *Tr-MYB134* repress flavonoid biosynthetic genes.** Promoter activation/repression dual luciferase assays upon **(A)** the *Tr-DFR* (anthocyanin and proanthocyanidin biosynthetic gene) promoter or **(B)** the *Tr-ANR* promoter (proanthocyanin biosynthetic gene), using *Agrobacterium*-infiltrated *Nicotiana benthamiana* leaves. Combinations of activator R2R3-MYB genes Tr-RED LEAF (anthocyanin) or Ta-MYB14 (proanthocyanidin) were tested with or without the bHLH factor Tr-AN1, and the R2R3-MYB repressors Tr-MYB133 or Tr-MYB134. Firefly luciferase activity was normalized to Renilla luciferase activity (LUC/REN); mean ± SEM *n* = 5 biological replicates are shown. Mean that are significantly different are indicated by different letters, as determined by *post hoc* Fisher’s LSD (5%).

The *DFR* promoter was activated when the R2R3-MYB anthocyanin regulator *Tr-RED LEAF* was co-infiltrated (**Figure 2A**), presumably forming MBW complexes with endogenous WDR and bHLH proteins (bHLH1 clade/JAF13) that are expressed in *Nicotiana* leaves (Albert et al., 2014a; Montefiori et al., 2015). Co-expression of *Tr-AN1* (bHLH2 clade) enhanced the activation. However, co-transformation with *Tr-MYB133* or *Tr-MYB134* reduced the activity of the *DFR* promoter. Similarly, the R2R3-MYB proanthocyanidin regulator *Ta-MYB14* activated the *DFR* promoter when the bHLH *Tr-AN1* was coinfiltrated, but this was repressed by *Tr-MYB133* and *Tr-MYB134*. The *ANR* promoter was activated when *Ta-MYB14* was coinfiltrated with *Tr-AN1*, but this was repressed by *Tr-MYB133* and *Tr-MYB134* (**Figure 2B**). The anthocyanin regulator *Tr-RED LEAF* was unable to activate the *ANR* promoter, even when the bHLH *Tr-AN1* was coinfiltrated.

### Tr-MYB133 and Tr-MYB134 Participate in Gene Regulation Networks

The expression patterns for *Tr-MYB133, Tr-MYB134* and anthocyanin/proanthocyanidin biosynthesis and regulatory genes were examined to establish whether these genes are associated with the accumulation of these metabolites. This was conducted using *T. repens* germplasm and tissues that accumulate anthocyanins or proanthocyanidins, due to the expression of endogenous anthocyanin-related R2R3-MYB genes (e.g., *Tr-RED LEAF*), or the proanthocyanidin regulator *Tr-MYB14*, respectively.

**FIGURE 3:**
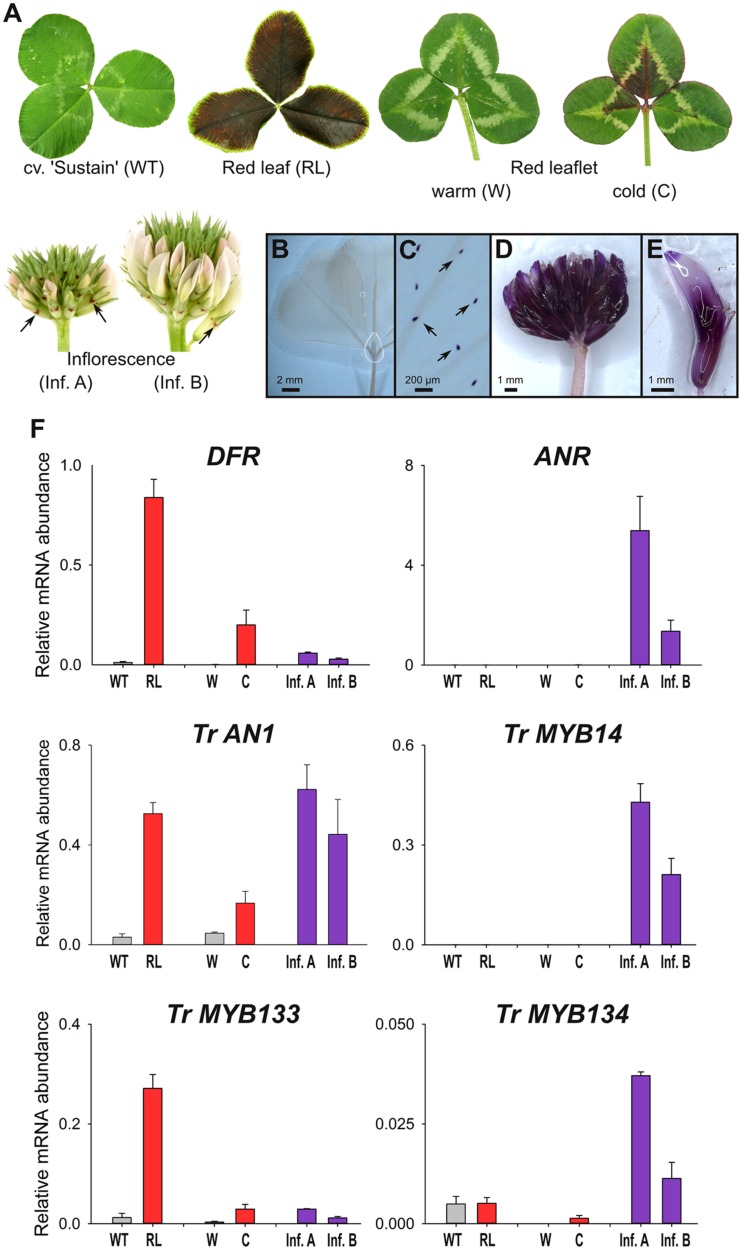
***MYB133* and *MYB134* are expressed in tissues accumulating anthocyanin and proanthocyanidins.** **(A)** Anthocyanin pigmentation phenotypes of samples used for qRT-PCR. White clover ‘Sustain’ (WT) lacks anthocyanins in leaves, while a genotype with the ‘Red leaf’ (RL) anthocyanin mark accumulates anthocyanins throughout the lamina. A genotype with the ‘Red leaflet’ trait lack anthocyanins when grown under warm conditions (W), but are induced with cold temperature (C). Inflorescences have low quantities of anthocyanins, although anthocyanin spots (arrows) are present on the calyx. (**B–E)** Tissues stained with DMACA to detect proanthocyanidins (purple). **(B)** White clover leaves lack proanthocyanidins, except in their trichomes **(C)**. **(D)** Proanthocyanidins accumulate in inflorescences and **(E)** within individual florets. **(F)** Relative transcript abundance of genes involved in anthocyanin and proanthocyanidin synthesis and regulation was determined by qRT-PCR. Mean ± SEM *n* = 4 biological replicates is shown.

Wild-type plants from cultivar ‘Sustain’ lack anthocyanin leaf markings (**Figure 3A**). In contrast, *T. repens* with ‘red leaf’ patterning have dark red leaves due to the accumulation of anthocyanins, which is regulated by the R2R3-MYB gene *RED LEAF_RL_* (Albert et al., 2015). Clonal copies of a *T. repens* genotype with the ‘red leaflet’ (distinct from ‘red leaf’) anthocyanin leaf marking were grown in a greenhouse (warm), or outside during winter (cold). Anthocyanin pigmentation associated with the ‘red leaflet’ locus (*Vrl*) only occurs in response to cold temperatures (**Figure 3A**). However, the R2R3-MYB activator responsible for ‘red leaflet’ patterning has not yet been conclusively identified or mapped to the *Vrl* locus (Albert et al., 2015). Developing inflorescences contain very little anthocyanin, and petals appear white or pale pink, although anthocyanin spots are present on the calyx.

The presence and spatial localization of proanthocyanidins was determined by staining tissues with DMACA, which reacts with polymerized flavanols to form an insoluble blue/purple precipitate. DMACA does not react with anthocyanin or other flavonoids (e.g., flavonols). Proanthocyanidins do not accumulate to high levels in white clover leaves (including high-anthocyanin genotypes; **Figure 3B**), although upon close examination DMACA staining is observed in trichomes (**Figure 3C**; see also Hancock et al., 2012). In contrast, inflorescences contain proanthocyanidins and stain strongly with DMACA (**Figures 3D,E**; see also Abeynayake et al., 2012).

Transcript abundance for genes involved in anthocyanin and proanthocyanidin biosynthesis and regulation was determined by quantitative RT-PCR (**Figure 3F**). Transcript abundance for *DIHYDROFLAVONOL-4 REDUCTASE* (*DFR*), a biosynthetic gene common to anthocyanin and proanthocyanidin pathways, strongly correlated with the presence of anthocyanins; the highest transcript levels were detected in the high-anthocyanin genotype (RL), and the ‘red leaflet’ plants exposed to cold (C) had ∼120-fold higher transcript levels for *DFR* than plants grown under glasshouse conditions (W). *DFR* transcripts were also detected in developing inflorescences at modest levels. By contrast, transcripts for the proanthocyanidin biosynthetic gene *ANTHOCYANIDIN REDUCTASE* (*ANR*) were detected in developing inflorescences, particularly in immature buds (Inf. A), but were only detected at trace levels in leaves (**Figure 3F**). Transcript abundance for the bHLH factor *Tr-AN1* were elevated in leaf samples accumulating anthocyanin (RL, C) or in developing inflorescences, which accumulate proanthocyanidins. Transcripts for the proanthocyanidin regulator, *Tr-MYB14*, were abundant in developing inflorescences, particularly in immature buds (Inf. A), but were not detected in the leaf samples. *Tr-MYB133* expression correlated with the accumulation of anthocyanins, with high transcript abundance detected in leaves of the high-anthocyanin genotype (RL), and ‘red leaflet’ plants exposed to cold (C) had ∼9-fold higher transcript levels for *Tr-MYB133* than plants grown under glasshouse conditions (W). *Tr-MYB133* transcripts were detected in inflorescences at low levels. By contrast, *Tr-MYB134* expression was associated with proanthocyanidin accumulation, with highest transcript abundance detected in immature inflorescences. Trace levels of *Tr-MYB134* were detected in leaf samples.

A transgenic white clover line expressing *Ta-MYB14* from a *CaMV35S* promoter (Hancock et al., 2012) was also analyzed (**Supplementary Figure S2**). This line exhibits the ‘red midrib’ and ‘red V’ anthocyanin leaf markings, and thus anthocyanin-related R2R3-MYB genes are also expressed in leaves (Albert et al., 2015). *Tr-MYB134* was highly expressed in leaves ectopically expressing *Ta-MYB14*, while transcript abundance of *Tr-AN1* was not significantly altered compared to controls.

**FIGURE 4:**
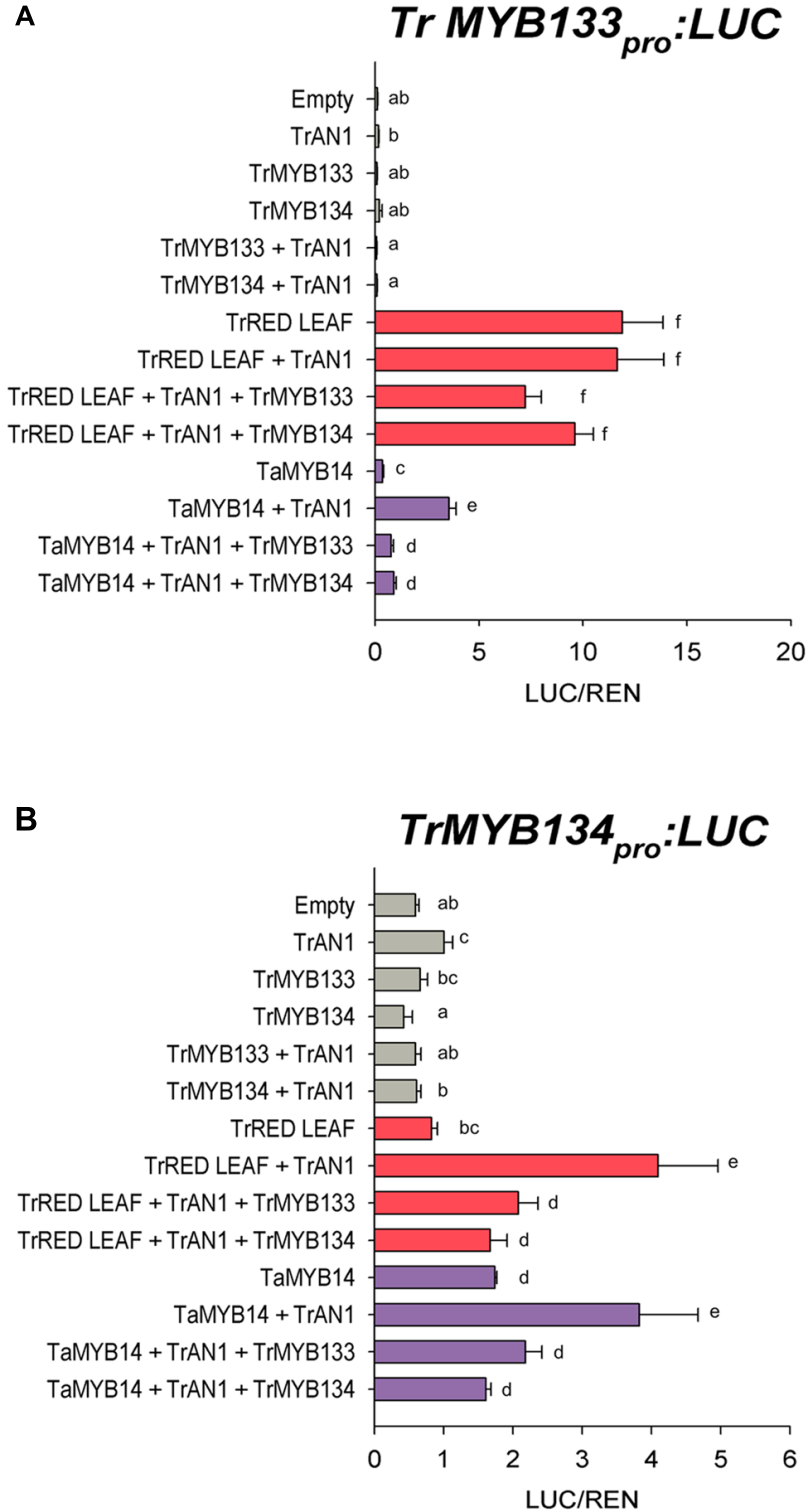
***Tr-MYB133* and *Tr-MYB134* are regulated by different MBW complexes.** Promoter activation/repression dual luciferase assays upon **(A)** the *Tr-MYB133* promoter or **(B)** the *Tr-MYB134* promoter, using *Agrobacteria*-infiltrated *N. benthamiana* leaves. Combinations of activator R2R3-MYB genes Tr-RED LEAF (anthocyanin) or Ta-MYB14 (proanthocyanidin) were tested with or without the bHLH factor Tr-AN1, and the R2R3-MYB repressors Tr-MYB133 or Tr-MYB134. Firefly luciferase activity was normalized to Renilla luciferase activity (LUC/REN); mean ± SEM *n* = 5 biological replicates are shown. Means that are significantly different are indicated by different letters, as determined by *post hoc* Fisher’s LSD (5%).

The expression patterns for *Tr-MYB133* and *Tr-MYB134* raised the possibility that these genes might be directly regulated by MBW complexes containing anthocyanin- or proanthocyanidin-specific R2R3-MYB activators, respectively. The ability for the anthocyanin R2R3-MYB regulator Tr-RED LEAF and the proanthocyanidin regulator Ta-MYB14 to regulate *Tr-MYB133* and *Tr-MYB134* was investigated by promoter activation assays. The promoters of *Tr-MYB133* and *Tr-MYB134* were isolated and cloned into a dual luciferase reporter construct. *Tr-RED LEAF* strongly activated the *Tr-MYB133* promoter (acting with endogenous bHLH and WDR proteins), even without the addition of the bHLH partner *Tr-AN1* (**Figure 4A**). Co-infiltration with *Tr-MYB133* weakly repressed this activation. *Ta-MYB14* only weakly activated the *Tr-MYB133* promoter and required the co-infiltration of the bHLH *Tr-AN1*. Co-infiltration with either *Tr-MYB133* or *Tr-MYB134* strongly repressed the activation by *Ta-MYB14*.

The *Tr-MYB134* promoter was responsive to the activity of *Ta-MYB14*, which was enhanced by the addition of *Tr-AN1* (**Figure 4B**). *Tr-RED LEAF* did not activate the *Tr-MYB134* promoter unless the bHLH factor *Tr-AN1* was co-infiltrated, and this was repressed by either *Tr-MYB133* or *Tr-MYB134*. However, *Ta-MYB14* alone did activate the *Tr-MYB134* promoter, and was enhanced with the addition of *Tr-AN1*. Co-infiltration with *Tr-MYB133* or *Tr-MYB134* repressed the activation by *Ta-MYB14*. The promoter activation assays agreed with the qPCR data, with the *Tr-MYB133* promoter being more responsive to anthocyanin regulators, while the *Tr-MYB134* promoter was more responsive to the proanthocyanidin regulator Ta-MYB14.

## Discussion

In all angiosperm species studied to date, anthocyanin and proanthocyanidin biosynthesis is regulated by MBW complexes. The current understanding of how anthocyanins and proanthocyanidins are regulated has recently been extended to include the activities of repressive R2R3- and R3-MYB proteins, which operate within a conserved gene-regulation network (Dubos et al., 2008; Matsui et al., 2008; Albert et al., 2014a,b). In this study, the roles of R2R3-MYB repressors for regulating anthocyanin and proanthocyanidin biosynthesis were investigated in the forage legume, white clover. Two R2R3-MYB repressors, which are conserved in model legumes, were shown to repress the activity of anthocyanin and proanthocyanidin MBW complexes. These two repressors have subfunctionalized in terms of their regulation, providing a mechanism that allows anthocyanin and proanthocyanidin synthesis to be regulated separately.

### Two Clades of Repressors in Legumes are Associated with Proanthocyanidin and Anthocyanin Regulation

Within the available sequence databases for *M. truncatula*, *L. japonicus* and *G. max*, two distinct R2R3-MYB genes exist that encode putative repressors of anthocyanin and/or proanthocyanidin biosynthesis in legumes. Using a candidate-gene approach, orthologs of these two genes were isolated from the forage legume, *T. repens*. These genes form two distinct clades when analyzed with other R2R3-MYB repressors, but share the bHLH interaction domain and EAR active repression motif, which are necessary for active R2R3-MYB repressors to assert their repressive activity (Aharoni et al., 2001; Albert et al., 2014a). These two genes are located on different linkage groups in *Medicago* and *Glycine*, and it is likely this will also be the case in *Lotus* and *Trifolium*, although data are currently not available for these genera [*Lj-MYB133* is not present in the current genome assembly of *L. japonicus* (Shelton et al., 2012), and the *T. repens* genome is not available]. The conservation and retention of these two genes in legumes suggests that they may have non-redundant functions.

Tr-MYB133 and Tr-MYB134 have similar repressive activities, but based upon the transcript abundance patterns have different roles regulating flavonoid synthesis. In luciferase assays, Tr-MYB133 and Tr-MYB134 both repressed the promoters of the flavonoid genes *DFR* (common to anthocyanin and proanthocyanidin synthesis) and *ANR* (proanthocyanidin-specific; **Figure 2**). Interestingly, they did not exhibit specificity for the MBW complexes they acted upon, repressing complexes containing the R2R3-MYB activators Tr-RED LEAF (anthocyanin) and Ta-MYB14 (proanthocyanidin). While these two transcription factors have similar repressive activity, their expression patterns differ. The expression of *Tr-MYB133* was strongly associated with the accumulation of anthocyanin pigments, while *Tr-MYB134* expression was tightly associated with proanthocyanidin synthesis (**Figure 3**). This suggested that *Tr-MYB133* and *Tr-MYB134* have subspecialized to predominantly regulate anthocyanins and proanthocyanidins, respectively.

Subspecialization of R2R3-MYB repressors for anthocyanins vs. proanthocyanidins has yet to be demonstrated conclusively in other groups of plants, although several observations suggest this is likely to occur. In both grape (*Vitis vinifera*) and strawberry (*Fragaria* sp.), proanthocyanidins are synthesized at early stages of fruit development, while anthocyanins are produced during the late stages of ripening (Schaart et al., 2012; Huang et al., 2014). In grape, the R2R3-MYB repressor *Vv-MYBC2-L1* has a bi-phasic expression pattern that correlates with the synthesis of both proanthocyanidins (early) and anthocyanins (late) during berry development (Huang et al., 2014). In contrast, *Vv-MYBC2-L3* expression is associated with proanthocyanidin accumulation, and not anthocyanins (Cavallini et al., 2015). In strawberry, however, *Fa-MYB1* expression is associated with anthocyanins and not proanthocyanidins (Aharoni et al., 2001; Lin-Wang et al., 2010; Schaart et al., 2012). Thus, it appears that R2R3-MYB repressor gene family members have subspecialized for proanthocyanidin or anthocyanin biosynthesis in grape and strawberry, and it is anticipated that this may also occur in more diverse species.

The association of *Tr-MYB133* with anthocyanin synthesis, and *Tr-MYB134* with proanthocyanidin synthesis may occur because these genes are themselves directly regulated by pathway-specific MBW complexes. In petunia, the R2R3-MYB repressor *Ph-MYB27* is targeted by the MBW complexes that regulate anthocyanin synthesis to provide feedback repression, and it is proposed that this is conserved across eudicots (Albert et al., 2014a). This was examined by promoter activation assays upon the promoters of *Tr-MYB133* and *Tr-MYB134*, using the anthocyanin R2R3-MYB Tr-RED LEAF (Albert et al., 2015) and the proanthocyanidin R2R3-MYB Ta-MYB14 (Hancock et al., 2012). These assays agreed with the gene expression data (**Figures 3** and **4**), demonstrating that the *Tr-MYB133* promoter is more effectively activated by MBW complexes that contain an anthocyanin-related R2R3-MYB, compared to the proanthocyanidin regulator.

The *Tr-MYB134* promoter was activated by MBW complexes containing either the anthocyanin- or proanthocyanidin-related R2R3-MYBs. Interestingly, Tr-RED LEAF was unable to activate the *Tr-MYB134* promoter without the addition of Tr-AN1, which differs to the activation observed for the *Tr-MYB133* promoter. The findings from the promoter activation assays appear to contrast slightly with the gene expression studies. White clover plants with the ‘red leaf’ anthocyanin leaf marking ectopically express the *Tr-RED LEAF* MYB gene (Albert et al., 2015), yet express *Tr-MYB134* poorly (**Figure 3**). However, in tissues expressing *Tr-MYB14* and accumulating proanthocyanidins, *Tr-MYB134* was expressed. Similar observations have been made in *Medicago*, where ectopic expression of the proanthocyanidin regulators *Mt-MYB5* and *Mt-MYB14* resulted in 80- and 10-fold increases, respectively, in the expression of Medtr5g079670, the ortholog of *Tr-MYB134* (Liu et al., 2014). This suggests that *Tr-MYB134* is normally regulated by the proanthocyanidin-related R2R3-MYB factors, and this is conserved in other legumes.

### MYB-bHLH-WDR Gene Regulation Networks Control Anthocyanin and Proanthocyanidin Synthesis in Legumes

Recently, a multi-species model was proposed for the MBW complex and the gene regulation networks that they operate within, integrating the activities of both activator and repressor transcription factors (Albert et al., 2014a). While this model was proposed for anthocyanin regulation, this also has implications for proanthocyanidin regulation, since anthocyanin and proanthocyanidin regulation occurs by a similar mechanism and shares MBW components. Key features of the models include (i) R2R3-MYB repressors are expressed in tissues to prevent ectopic synthesis of anthocyanins/proanthocyanidins; (ii) feedback repression occurs by both R2R3-MYB proteins containing active repression domains (EAR, TLLLFR), and by the mobile competitive R3-MYB proteins; (iii) MBW complexes can contain multiple MYB proteins (e.g., MYB activator + MYB repressor), bridged by dimerized bHLH proteins; (iv) hierarchical activation of the AN1-clade bHLH factor occurs to provide reinforcement.

Many commonalities exist between the proposed model (Albert et al., 2014a) and the findings from this study and with data from other legume species, although there are some interesting differences. While the core features of the MBW complex are highly conserved in legumes—including the requirement for the bHLH2/AN1/TT8 clade bHLH and WDR proteins for anthocyanin and proanthocyanidin regulation (Pang et al., 2009; Hellens et al., 2010; Verdier et al., 2012) and MBW complex assembly (Liu et al., 2014)—the involvement of MYB repressors differ. It was anticipated that *Tr-MYB133* and *Tr-MYB134* might be expressed highly in leaves to prevent ectopic accumulation of anthocyanins and proanthocyanidins. However, *Tr-MYB133* and *Tr-MYB134* were only expressed highly in tissues accumulating anthocyanins or proanthocyanidins, respectively, and do not appear to have roles preventing ectopic accumulation of these metabolites. This contrasts with petunia and *Arabidopsis*, where the R2R3-MYB repressor *Ph-MYB27* and (truncated R2) R3-MYB repressor *At-MYBL2* are expressed highly in leaves under non-stress conditions, while exposure to light-stress results in a dramatic reduction of expression (Dubos et al., 2008; Albert et al., 2011). This de-repression is likely to be important to allow plants to respond to changing environmental conditions, where the accumulation of anthocyanins is advantageous (e.g., to ameliorate light stress). Thus, the loss of the repressors *Ph-MYB27* or *At-MYBL2* results in enhanced accumulation of anthocyanins in petunia and *Arabidopsis*, respectively (Dubos et al., 2008; Albert et al., 2014a). It is unlikely that losing MYB133 or MYB134 activity in clover or other legumes (e.g., *Medicago*) will significantly alter the distribution of anthocyanins or proanthocyanidins, although it is anticipated the anthocyanin/proanthocyanidin content would increase in tissues that already accumulate these compounds. The difference in the regulation of MYB133 and MYB134 compared to repressors from other species raises questions about how legumes prevent ectopic accumulation of anthocyanins/proanthocyanidins and whether these activities are performed by additional repressive MYB family members, or by other transcription factors.

**FIGURE 5:**
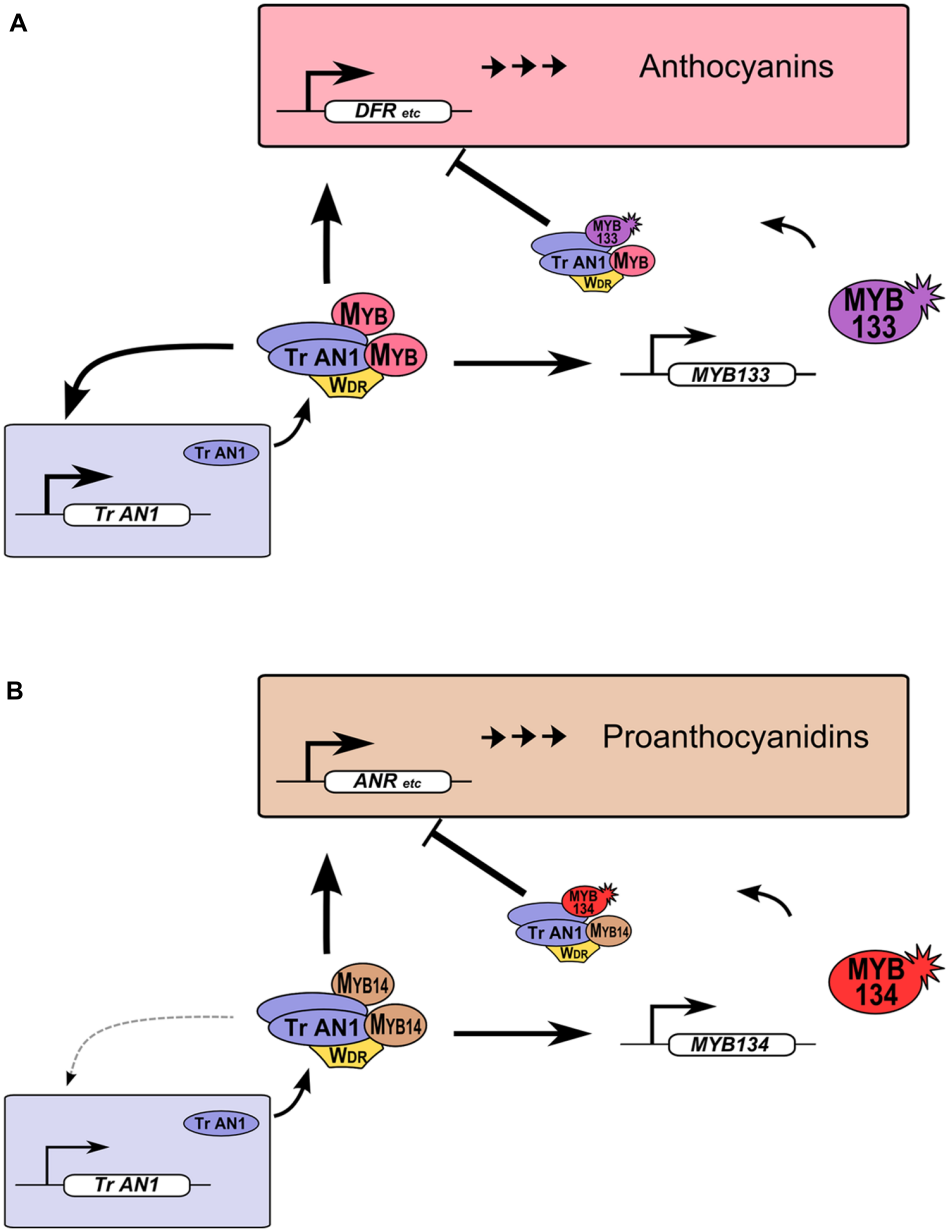
**Tr-MYB133 and Tr-MYB134 participate in MBW regulatory networks.** **(A)** Anthocyanin MBW gene regulation network. MBW complexes containing anthocyanin R2R3-MYB activate genes required for anthocyanin biosynthesis and transport, resulting in anthocyanin pigment accumulation. The AN1/TT8 clade bHLH (*Tr-AN1*) is also activated, to provide reinforcement, while *Tr-MYB133* is activated to provide feedback repression upon the MBW complex. **(B)** The proanthocyanidin MBW gene regulation network activates genes required for proanthocyanidin biosynthesis and transport, and *Tr-MYB134* to provide feedback repression. Proanthocyanidin MBW activation complexes may include other MYB activators, such as MYB5 or PAR (not shown). The AN1/TT8 clade bHLH gene does not appear to be a high affinity target of MYB14.

R2R3-MYB proteins provide feedback repression to MBW gene regulation networks in legumes (**Figure 5**). This feature of MBW gene regulation networks was observed in petunia and proposed to be conserved in eudicots. Such feedback regulation likely allows for fine-tuning of gene expression (Albert et al., 2014a; Cavallini et al., 2015). We have demonstrated that this also occurs for Tr-MYB133 and Tr-MYB134 in white clover, but these two genes have subspecialized to provide feedback repression for anthocyanin (**Figure 5A**) and proanthocyanidin biosynthesis (**Figure 5B**), respectively. These two repressors have the amino acid motif that is required to bind bHLH proteins, contain EAR repression motifs in their C-termini, and are from the Ph-MYB27/Fa-MYB1 clade of R2R3-MYB repressor (**Figure 1**). Thus, it is anticipated that Tr-MYB133 and Tr-MYB134 will be incorporated into MBW complexes, and recruited to promoters by activator R2R3-MYB proteins, as occurs for Ph-MYB27 (Albert et al., 2014a). It remains to be determined if competitive R3-MYB proteins also provide feedback repression upon anthocyanin and proanthocyanidin synthesis in legumes, as occurs in petunia and *Arabidopsis* (Zhu et al., 2009; Albert et al., 2014a).

The activation of genes encoding R2R3-MYB repressors by MBW complexes appears to be widely conserved in eudicots. In petunia, ectopic expression of anthocyanin MYBs (Ph-DPL, Ph-PHZ) resulted in elevated expression of the repressor *Ph-MYB27*, and this was shown to occur by direct activation upon the *Ph-MYB27* promoter by anthocyanin MBW complexes (Albert et al., 2014a). Similarly, ectopic expression of the anthocyanin MYB *Vl-MYBA1* (Cutanda-Perez et al., 2009) or the proanthocyanidin MYBs *Vv-MYBPA1* or *Vv-MYBPA2* (Terrier et al., 2009) in grape cultures resulted in ectopic expression of *Vv-MYBC2-L1*. This agrees with the temporal expression pattern for *Vv-MYBC2-L1*, which correlates with both proanthocyanidin and anthocyanin synthesis during berry development (Huang et al., 2014). Interestingly, while the temporal expression of *Vv-MYBC2-L3* suggests it may be associated with proanthocyanidin regulation (Cavallini et al., 2015), its expression was not elevated in grape cultures expressing *Vv-MYBPA1/Vv-MYBPA2* (Terrier et al., 2009). It is not yet known if the apparent sub-specialization of particular R2R3-MYB repressor genes for anthocyanin or proanthocyanidin synthesis in grape (Vv-MYBC2-L3 – proanthocyanidins) or strawberry (Fa-MYB1 – anthocyanins; Aharoni et al., 2001; Schaart et al., 2012; ; Huang et al., 2014; Cavallini et al., 2015) occurs because they are regulated by distinct MBW complexes, or whether they regulated by developmental signals during fruit development and ripening.

The anthocyanin MBW complexes activate the expression of the *Tr-AN1/Mt-TT8* bHLH genes. The ‘red leaf’ anthocyanin leaf marking in white clover occurs because the R2R3-MYB gene *Tr-RED LEAF* is ectopically expressed (Albert et al., 2015), which results in enhanced expression of *Tr-AN1* (**Figure 3**). Similarly, *Medicago* plants expressing the anthocyanin R2R3-MYB gene *Mt-LAP1* have enhanced expression of *Mt-TT8* (Peel et al., 2009). Thus, the reinforcement of bHLH expression by MBW complexes containing anthocyanin R2R3-MYB genes also occurs in legumes, as it does in petunia and *Arabidopsis* (Baudry et al., 2006; Albert et al., 2014a). Interestingly, it is less convincing that the proanthocyanidin regulator MYB14 is a strong activator of the AN1/TT8 bHLH genes. Ectopic expression of *Mt-MYB14* in *Medicago* only increased the expression of *Mt-TT8* ∼2-fold (Liu et al., 2014), and overexpression of *Ta-MYB14* in white clover did not significantly enhance *Tr-AN1* expression (**Supplementary Figure S2**). Thus, *Tr-AN1/Mt-TT8* may be a lower affinity target of MYB14, and these bHLH genes may be primarily regulated by developmental signals. Alternatively, they may be regulated by other proanthocyanidin-related R2R3-MYB genes that are present in legumes (e.g., Mt-MYB5, Mt-PAR), or by MBW complexes that contain more than one type of proanthocyanidin MYB (Verdier et al., 2012; Liu et al., 2014).

The models presented in this study (**Figure 5**) build upon the genetic and molecular data that supports the existence of MBW complexes for regulating both anthocyanins and proanthocyanidins in legumes (Pang et al., 2009; Peel et al., 2009; Hellens et al., 2010; Verdier et al., 2012; Liu et al., 2014; Albert et al., 2015), integrating the activities of R2R3-MYB repressors. The use of transient assays in *N. benthamiana* to investigate the activity of MBW genes, including *MYB133* and *MYB134*, has been instrumental in overcoming the challenges posed by white clover – a species that is recalcitrant to molecular and genetic analysis (outcrossing, allotetraploid). The major limitation with such assays are that endogenous bHLH (bHLH1) and WDR proteins are expressed (Albert et al., 2014a; Montefiori et al., 2015), which can sometimes obscure the essential roles of the bHLH and WDR components. However, mutants for these MBW components in legumes completely lack anthocyanins and proanthocyanidins, such as Mendel’s classic ‘*A*’ (bHLH2) and ‘*A2*’ (WDR) genes in pea (Hellens et al., 2010) and *Mt-WDR1* in *Medicago* (Pang et al., 2009), which highlights the essential role of these proteins for MBW complex activity. It is anticipated that the models presented in this study will be further strengthened by the analysis of mutants for MBW components and the MYB repressors in *Medicago*.

## Conclusion

Two R2R3-MYB genes encoding flavonoid repressors are conserved in legumes, operating within the MBW gene regulation networks that control anthocyanin and proanthocyanidin synthesis. The MBW gene regulation network identified in white clover fulfills many of the key features proposed to be conserved in eudicots. This includes the involvement of R2R3-MYB repressors to provide feedback repression upon the MBW activation complexes and reinforcement in the expression of the bHLH factor. However, Tr-MYB133 and Tr-MYB134 are regulated by distinct MBW complexes, associated with anthocyanin and proanthocyanidin synthesis, respectively. This subspecialization provides a mechanism that allows for anthocyanin and proanthocyanidins to be regulated separately.

## Author Contributions

NA designed the study, performed experimentation, analyses, and wrote the manuscript.

## Conflict of Interest Statement

AgResearch and Grasslanz Technology Limited hold a patent for the commercial use of *Ta-MYB14* and related sequences. The reviewer José Tomás Matus and handling Editor David Caparros-Ruiz declared their shared affiliation, and the handling Editor states that, nevertheless, the process met the standards of a fair and objective review.

## Abbreviations

MBW: MYB-BHLH-WDR
