# Supplementary material for: Subspecialization of R2R3-MYB Repressors for Anthocyanin and Proanthocyanidin Regulation in Forage Legumes
Source: Front Plant Sci. 2015 Dec 23;6:1165. doi: 10.3389/fpls.2015.01165 (PMC4689181; doi:10.3389/fpls.2015.01165)
Supplement: Supplementary file 1 [file Data_Sheet_1.PDF]

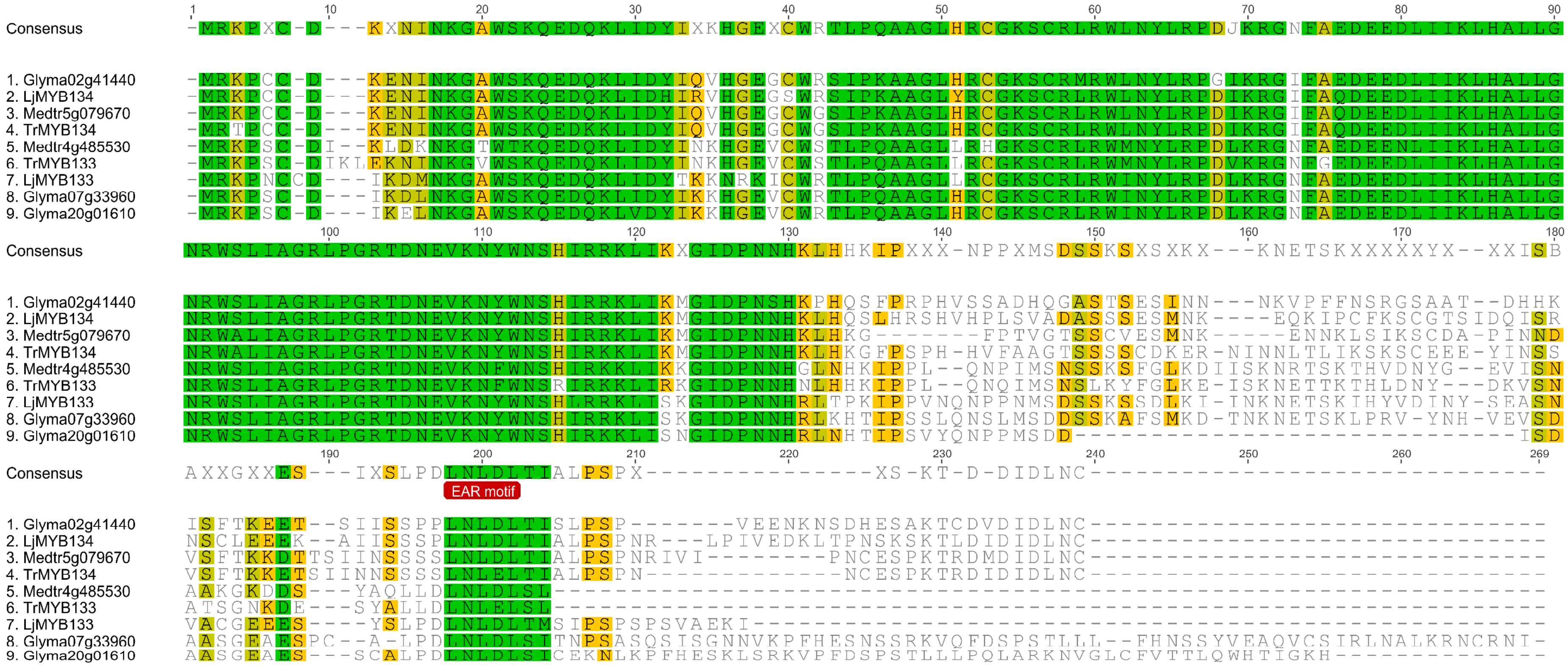

Figure S1 Amino acid alignment of putative anthocyanin/proanthocyanidin R2R3-MYB repressors from legume species

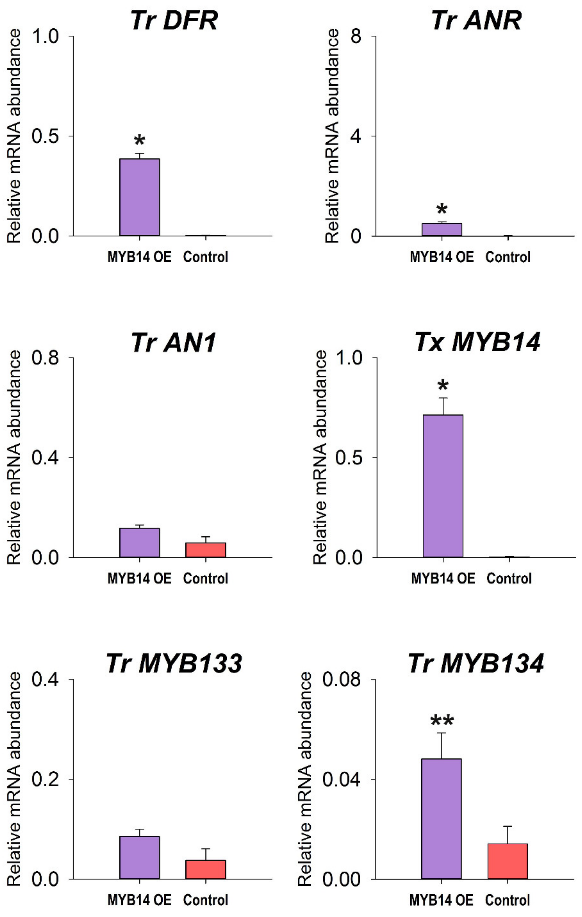

Figure S1

Figure S2 *Tr-MYB134* is induced in white clover plants ectopically expressing *Ta-MYB14*

The expression of genes involved in anthocyanin and proanthocyanidin synthesis and regulation were analysed in white clover plants expressing the proanthocyanidin regulator *Ta-MYB14* from a *CaMV35S* promoter (MYB14 OE), and plants that have silenced the transgene (control). Transcript abundance was determined by qRT-PCR and is expressed relative to the *ACTIN*, *GAPDH* and *PP2*. *Tx-MYB14* indicates the assay detects both the endogenous *Tr-MYB14* transcripts in *Trifolium repens*, and those derived from the *Ta-MYB14* transgene (*T. arvense*). Means ± SEM *n*=4 biological replicates are shown. Means that are significantly different (p<0.01) are indicated (*), determined by *t*-test. *MYB134* transcript levels are approaching significance (p= 0.075, **).

Table S1 Primer and probe sequences

Primer name	S/AS	Gene/purpose	Sequence
NAg3	S	Degenerate R2R3 MYB repressor	ATGAGRAARCCTWGTTGTGA
NAg4	AS	Degenerate R2R3 MYB repressor	TGAGAATTCCAATARTTYTTCAC
NAg11	AS	3’RACE cDNA synthesis	GCTCGCGAGCGCGTTTAAACGCGCACGCGTTTTTTTTTTTTTTTTTTVN
NAg12	AS	3’RACE1	GCTCGCGAGCGCGTTTAAAC
NAg13	AS	3’RACE2	GCGTTTAAACGCGCACGCGT
NAg34	S	Tr MYB133 3’RACE1	AAGCTTCATGCACTTCTAGGC
NAg35	S	Tr MYB133 3’RACE2	CACTAATTGCTGGGAGATTGC
NAg45	AS	Degen R2R3 MYB repressor/5’RACE	TTGGATCAATTCCCATYTTTATTAG
NAg46	AS	Degen R2R3 MYB repressor/5’RACE	CAGGCAACCTTCCAGCWATGA
NAg47	AS	Tr MYB133 5’RACE	CACCGAAGTAGACCTGCAGCTTGAG
NAg49	AS	Tr MYB133 5’RACE	CATTAAGAAGTTTGACGTACGG
NAg72	S	Tr MYB134 5’UTR/3’RACE	AGGGTAACCACTCACAAGTAC
NAg73	S	Tr MYB134 ATG/ 3’RACE	CACCAAAATGAGAACACCTTGTTGTGAC
NAg95	AS	Tr MYB134 Stop	TTAACAATTAAGATCAATATCTATATCC
NAg96	AS	Tr MYB134 3’UTR	GCGGTCCTATCAATTCTGATA
NAg100	S	Tr MYB133 5’UTR	ATCATCTCTCTATCTCTTGCTCTA
NAg101	S	Tr MYB133 ATG	CACCAAAATGAGGAAACCTAGTTGTGATATC
NAg102	AS	Tr MYB133 Stop	TCATAGTGAGAGCTCTAGATTTAA
NAg103	AS	Tr MYB133 3’UTR	TAAATGGAAGATTTCATTTATGRAAG
NAg151	S	Tr AN1 qPCR	GTTGCGTCGGTTCAAGTTTC
NAg152	AS	Tr AN1 qPCR	TCAATCCTCAACTCCCTCAAC
NAg161	S	Tr MYB133 qPCR	AGGTCTACTTCGGTGTGGT
NAg162	AS	Tr MYB133 qPCR	CCACCTGTTGCCTAGAAGTG
NAg165	S	Tr MYB134 qPCR	TCACAAGTTACACAAAGGTTTTCC
NAg166	AS	Tr MYB134 qPCR	TTGATTAATGTGAGGTTATTGATGTTTC
TR11	S	Tr GAPDH qPCR	TCCAGTATTGAACGGTAAATTGAC
TR12	AS	Tr GAPDH qPCR	TCTGATTCCTCCTTGATAGCAG
TR13	S	Tr PP2 qPCR	AAGCTGTTGGTCCTGATTCC
TR14	AS	Tr PP2 qPCR	CGGGAGAACTTAGTCACTTTCC

Gene	Probe	Primer S	Primer AS
MYB14	6FAM-ACCATGGAGCTTAATGTATTCAGTGAGGA ZEN	GCTTGAATAGAGGTGCTTGGACA	GCTCTTTTTGGAAGGTTTCTCC
DFR	6FAM-TGGATTTTGAGTCCAAGGACCCTGA ZEN	GGAGTTTTTCATGTTGCTACACC	GCTTTCATGATGTCTAGGACTCC
ANR	6FAM-TGCCTGTTATTAGAGACATTGCCAAGCC ZEN	GGTCCTTCTCTCACACCAGA	GCATTCCTTTCAGAGCATTTATGA
ACTIN	6FAM-TGCTGAGGGATGCAAGGATTGATCC ZEN	GAAGATTAAGGTTGTGGCTCCA	GATATCCACATCTGCTGGAAGG

Table S2 Accession numbers for sequences used to construct phylogenetic tree

Sequence	Accession/gene ID
At-MYB16	AT5G15310
Ph-MYB1	CAA78386
Am-MIXTA	CAA55725.1
Lj-MYB7	Shelton et al. (2012) Plant Physiology 159, 531-547.
Tr-MYB7	KT699107
At-MYB4	AT4G38620
Ph-MYB4	ADX33331.1
Lj-MYB4	Shelton et al. (2012) Plant Physiology 159, 531-547.
Medtr4g073420.1	Medtr4g073420.1
Tr-MYB4	KT699106
Md-MYB16	XP_008363293
Vv-MYB4a	ABL61515.1
Vv-MYB4b	ACN94269.1
Md-MYB17	ADL36757.1
Vv-MYBC2-L2	ACX50288.2
Fa-MYB1	AF401220_1
Ph-MYB27	AHX24372
Lj-MYB133	Shelton et al. (2012) Plant Physiology 159, 531-547.
Glyma07g33960.1	Glyma07g33960.1
Glyma20g01610.1	Glyma20g01610.1
Medtr4g485530.1	Medtr4g485530.1
Tr-MYB133	KT699108
Pt-MYB165	XP_002315890.2
Pt-MYB194	XP_002311495.2
Lj-MYB134	Shelton et al. (2012) Plant Physiology 159, 531-547.
Glyma02g41440.1	Glyma02g41440.1
Medtr5g079670	Medtr5g079670
Tr-MYB134	KT699109
Vv-MYBC2-L3	AIP98385.1
Md-MYB111	ADL36754.1
Pt-MYB182	XP_002305872.1
Vv-MYBC2-L1	AFX64995.1
Sl-MYB12	EU419748.1
Gt-MYBP3	AB733016.1
At-MYB12	AT2G47460
Sb-Yellow seed1	AAX44239.1
Zm-P1	NP_001278607.1
At-MYB5	AT3G13540
Mt-MYB5	XM_003601561.1
Vv-MYB5b	AAX51291
At-TT2	AT5G35550
Lj-TT2a	BAG12893.1
Lj-TT2b	BAG12894
Lj-TT2c	BAG12895
Glyma13g16890	Glyma13g16890
Glyma17g05831	Glyma17g05831

To-MYB14	AFJ53052.1
Tr-MYB14	AFJ53050.1
Taf-MYB14	AFJ53046.1
Ta-MYB14	AFJ53053
Mt-MYB14	AFJ53057.1
Dk-MYB2	AB503699.1
Vv-MYBPA2	ACK56131
Fa-MYB10	ABX79947.1
Md-MYB10	EU518249.2
At-PAP1	AT1G56650
At-PAP2	AT1G66390
Ph-AN2	AAF66727.1
Ph-PHZ	ADW94951.1
Ph-DPL	ADW94950
Am-Venosa	ABB83828.1
Am-Rosea2	ABB83827.1
Mt-LAP2	ACN79539.1
Mt-LAP3	ACN79542.1
Tr-RED V-a	AIT76565.1
Tr-RED LEAF	AIT76557.1
Mt-LAP1	ACN79541.1
Mt-LAP4	ACN79540.1
Tr-RED LEAF DIFFUSE-a	AIT76556.1
Tr-RED LEAF DIFFUSE-b	AIT76560.1
